# Optimizing the Properties of InGaZnO_x_ Thin Film Transistors by Adjusting the Adsorbed Degree of Cs^+^ Ions

**DOI:** 10.3390/ma12142300

**Published:** 2019-07-18

**Authors:** He Zhang, Yaogong Wang, Ruozheng Wang, Xiaoning Zhang, Chunliang Liu

**Affiliations:** 1 Key Laboratory of Physical Electronics and Devices, Xi’an Jiaotong University, Ministry of Education, Xi’an 710049, China; 2 School of Electronic and Information Engineering, Xi’an 710049, China

**Keywords:** low-temperature fabrication, ions adsorption, IGZO TFTs, device performance

## Abstract

To improve the performance of amorphous InGaZnO_x_ (a-IGZO) thin film transistors (TFTs), in this thesis, Cs^+^ ions adsorbed IGZO (Cs-IGZO) films were prepared through a solution immersion method at low temperature. Under the modification of surface structure and oxygen vacancies concentrations of a-IGZO film, with the effective introduction of Cs^+^ ions into the surface of a-IGZO films, the transfer properties and stability of a-IGZO TFTs are greatly improved. Different parameters of Cs^+^ ion concentrations were investigated in our work. When the Cs^+^ ions concentration reached 2% mol/L, the optimized performance Cs-IGZO TFT was obtained, showing the carrier mobility of 18.7 cm^2^ V^−1^ s^−1^, the OFF current of 0.8 × 10^−10^ A, and the threshold voltage of 0.2 V, accompanied by the threshold voltage shifts of 1.3 V under positive bias stress for 5000 s.

## 1. Introduction

In the last decade, amorphous InGaZnO_x_ (a-IGZO) thin film transistors (TFTs) have been extensively researched due to their outstanding performance, including superior carrier mobility (*μ_FE_*), low subthreshold swing (*S.S*), large switching current radio (*I_ON_/I_OFF_*), and high transparency under visible light [1,2,3]. It showed great significance in the applications of next-generation electronic devices such as displays [4,5], sensors [6,7], and memories [8,9], especially in wearable and flexible devices like foldable displays and e-paper [10,11,12]. Since the wearable and flexible devices should be prepared onto a polymeric substrate which cannot afford the high temperature, the a-IGZO TFTs need to be prepared at a low temperature for use in wearable and flexible applications. 

In recent years, solution process and magnetron sputtering have been commonly used to achieve low temperature prepared a-IGZO TFTs. However, a-IGZO prepared by the solution process shows disadvantages such as lots of film defects, large roughness, and poor uniformity of large areas—inducing low carrier mobility of a-IGZO TFTs (generally < 10 cm^2^ V^−1^ s^−1^), which cannot satisfy the requirements of high-definition flexible display applications [13,14,15,16]. The studies also showed that most of the sputtered a-IGZO TFTs always need an annealing process to guarantee their high performance, since unannealed a-IGZO fabricated by magnetron sputtering still have many defects inside [17,18,19]. Although some scholars have fabricated a-IGZO TFTs by magnetron sputtering without annealing, the low mobility (<10 cm^2^ V^−1^ s^−1^) and stability (threshold voltage shifts > 5 V) of TFTs determined that they cannot afford the requirement of flexible devices [20,21,22]. Until now, it is still an open question to obtain a high-performance a-IGZO TFT at low temperature without an annealing process. 

Herein, a kind of Cs^+^ ion adsorption was used to modify the surface of a-IGZO film to improve the performance of a-IGZO TFTs. The excellent electrical properties of a-IGZO TFT were obtained under low-temperature conditions, without post-annealing, by adjusting the adsorption degree of Cs^+^ ions. Compared with traditional a-IGZO TFT fabricated by a high-temperature annealing process, excellent properties were observed in the optimized Cs^+^ ions adsorbed IGZO (Cs-IGZO) TFT. The high carrier mobility of 18.7 cm^2^ V^−1^ s^−1^, the low threshold voltage (*V_th_*) of 0.2 V, and the low *V_th_* shifts of 1.3 V under positive bias stress for 5000 s reveal the outstanding performance of the Cs-IGZO TFT.

## 2. Materials and Methods

**Figure 1:**
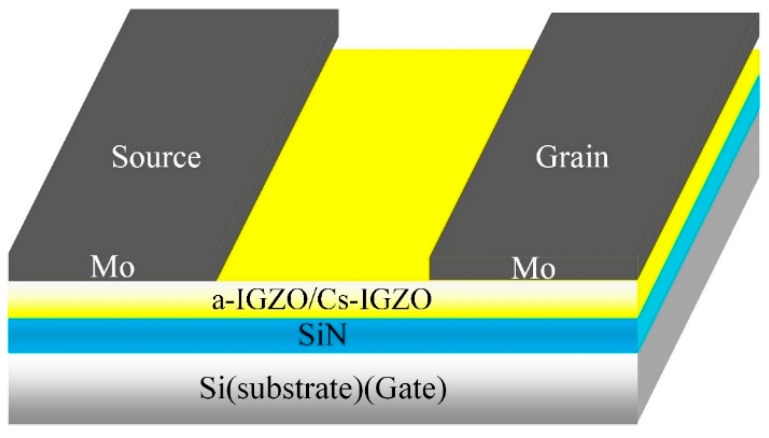
Structure of Cs^+^ ions adsorbed IGZO (Cs-IGZO) thin film transistor (TFT) and IGZO TFT.

The Bottom-gate staggered Cs-IGZO TFTs were fabricated and shown in Figure 1. In Cs-IGZO TFTs fabrication, heavily doped p-type silicon (P++-Si) was chosen to be the substrate and gate electrode. A layer of SiN_x_, with a thickness of 50 nm, was deposited on the Si wafer by plasma enhanced chemical vapor deposition (PECVD), serving as dielectric layer. Then, a-IGZO film with a thickness of 50 nm was deposited onto SiN_x_ by magnetron sputtering. The working pressure during a-IGZO film deposition was maintained at 5 mTorr with a mixture of Ar (24.3 sccm) 97% and O_2_ (0.7 sccm) 3% gases. The DC power and sputtering time were 80 W and 30 min, respectively. After IGZO deposition, the wet etching technique was applied to form active islands of TFTs. 

The Cs-IGZO film was formed by immersing the prepared substrate into CsHCO_3_ solution. To investigate the influence of the Cs^+^ ions concentration in the performance of Cs-IGZO film and TFTs, four different concentrations of CsHCO_3_ were prepared as shown in Table 1. The substrate was immerged into CsHCO_3_ solution for 60 min under 75 °C to ensure the sufficient adsorption of Cs^+^ ions into IGZO film. Then, Cs-IGZO films were cleaned by deionized water and dried in nitrogen. Finally, a 50 nm thick layer of molybdenum (Mo) was deposited through a shadow mask to form source (S) and drain (D) electrodes, which also defined the channel length as 500 μm.

Transfer properties and stability under positive gate bias of Cs-IGZO TFTs were measured by a semiconductor parameter analyzer (Keithley 4200SCS, Cleveland, OH, USA) in a dark box at room temperature. Hall mobility (*μ_Hall_*), carrier concentration, and resistivity of Cs-IGZO films were obtained by Hall Effect. The surface morphologies and the chemical composition of Cs-IGZO films were obtained by Atomic Force Microscope (AFM, INNOVA, Billerica, MA, USA), X-Ray Diffraction (XRD, D8 ADVANCE A25, Bruker, Karlsruhe, Germany), and X-ray Photoelectron Spectroscopy (XPS, Thermo Fisher ESCALAB Xi+, Waltham, MA, USA).

## 3. Results and Discussion

### 3.1. Electrical Characteristics of Cs-IGZO TFTs

**Figure 2:**
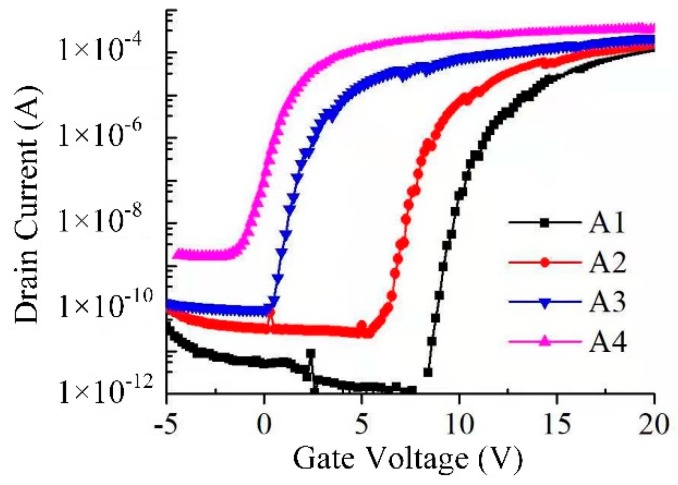
Transfer performance of sample A1 to A4.

The transfer properties of Cs-IGZO TFTs from sample A1 to A4 were measured and plotted in Figure 2. In the measurement of Cs-IGZO TFTs transfer properties, the drain–source voltage (*V_DS_*) is 10 V, with sweeping the source–gate voltage (*V_GS_*) from −5 to +20 V. *V_th_*, *μ_FE_*, and *S.S* of TFTs are extracted by the transfer properties of the TFTs and represented in Table 2, respectively. As observed with Figure 2 and Table 2, the *V_th_* of samples A1 to A4 gradually reduce from 7.9 V to −1.7 V, which indicates that the switching performance of Cs-IGZO TFTs can be adjusted with the changing of Cs^+^ ions concentration. The *μ_FE_* of samples A1 to A4 increase from 8.6 cm^2^ V^−1^ s^−1^ to 21.5 cm^2^ V^−1^ s^−1^, indicating that the transport speed of electrons of a-IGZO film can be significantly improved by increasing the Cs^+^ ions concentration. The *S.S* of samples A1 to A4 decreases from 0.28 V/decade to 0.22 V/decade, which means the defects inside the TFTs can be tapered by increasing the Cs^+^ ions concentration. In addition, the OFF current (*I_OFF_*) of samples A1 to A4 are calculated to be 5.6 × 10^−11^ A, 3.5 × 10^−10^ A, 0.8 × 10^−10^ A, and 6.7 × 10^−8^ A, which indicates that the drive characteristics of Cs-IGZO TFTs can be deteriorated by increasing the concentration of Cs^+^ ions.

In general, the oxygen molecules in air ambient can adsorb onto a-IGZO when a-IGZO is under positive bias and exposed in the atmosphere. The formation of oxygen adsorption is shown in the following equation:

(1) $$O_{2}\left(\mathrm{gas}\right)+e^{-}↔2O^{-}\left(\mathrm{solid}\right)$$

**Figure 3:**
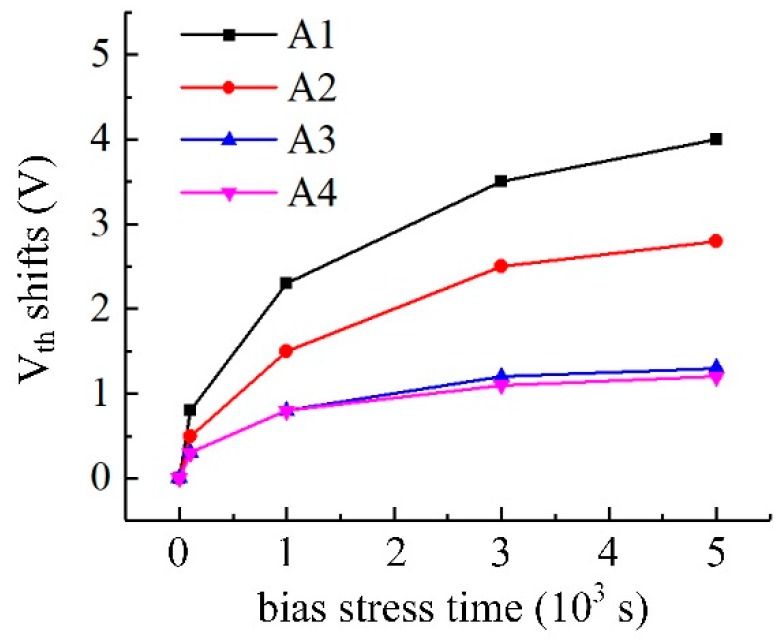
*V_th_* shift under positive bias stress of samples A1 to A4.

This adsorption of oxygen can change the physicochemical properties of the a-IGZO film, causing the *V_th_* positive shifts (Δ*V_th_*) of the a-IGZO TFT under working conditions, thereby reducing the stability of the IGZO TFT. Therefore, in order to investigate the effect of Cs^+^ ions adsorbed degree on the stability of Cs-IGZO TFTs, the Δ*V_th_* of samples A1 to A4 under positive bias (*V_GS_* = 10 V) for different time are calculated and represented in Figure 3, respectively. As observed with Figure 3, sample A1 has the largest Δ*V_th_* (3.9 V), represented by the black dash line. The Δ*V_th_* of samples A1 to A3 are gradually reduced, wherein the Δ*V_th_* of samples A2 (2.8 V) and A3 (1.3 V) are represented by red and blue lines, respectively. These results indicate the stability of Cs-IGZO TFTs can be optimized by increasing the Cs^+^ ions adsorbed degree of IGZO film. In addition, sample A4 has a similar Δ*V_th_* to sample A3 of 1.2 V, represented by the purple line, indicating that the stability of Cs-IGZO TFTs are no longer optimized by increasing the Cs^+^ ions adsorbed degree of IGZO film when the CsHCO_3_ concentration reached 2% mol/L. 

### 3.2. Surface Structure of Cs-IGZO Films

**Figure 4:**
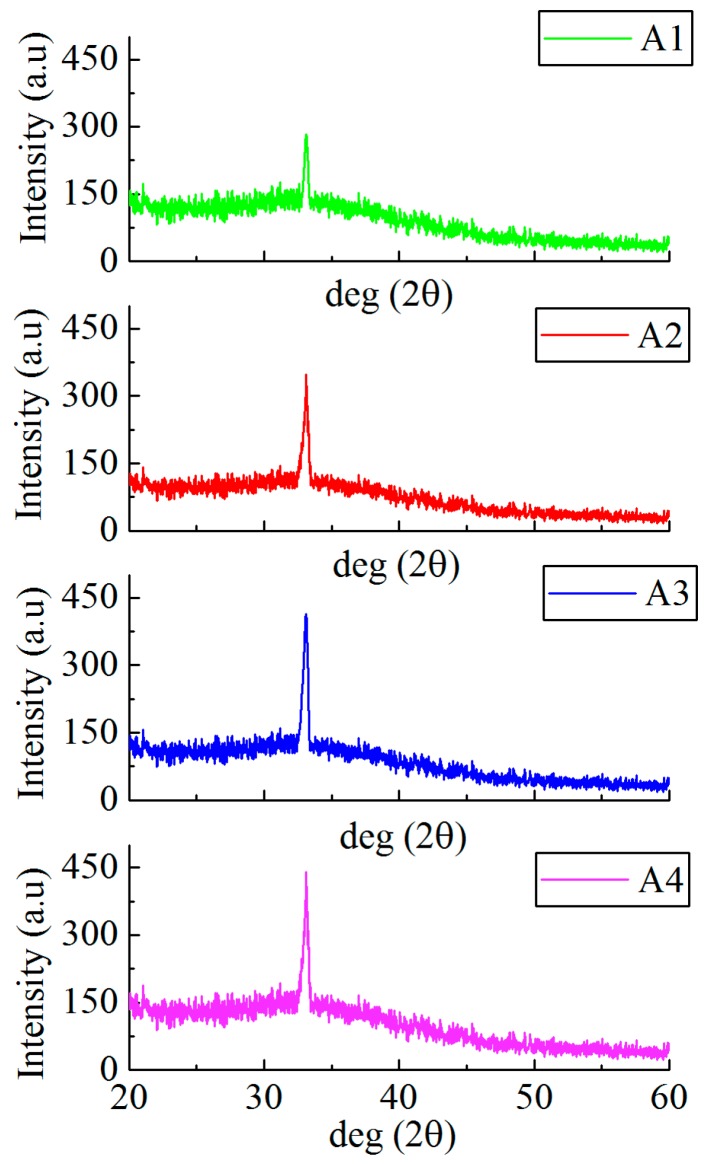
XRD results of samples of A1, A2, A3, and A4.

The surface crystal structures of samples A1 to A4 were measured by XRD, the results are shown in Figure 4.

As observed with Figure 4, the surfaces of the samples A1 to A4 all have a diffraction peak at a diffraction angle of 33.1°, indicating that the samples A1 to A4 all exhibit a single crystal state. The intensity of the diffraction peaks in samples A1 to A4 gradually increased, indicating that the surface crystallization strength of the Cs-IGZO film can be promoted by increasing the degree of adsorption of Cs^+^ ions, thereby obtaining the more regular surface structure of Cs-IGZO films. Since the diffraction peak at a diffraction angle of 33.1° of Cs-IGZO films corresponds to (101) orientation of indium, the XRD results of samples A1 to A4 also indicate that the regular-arranged In atoms are the primary cause of surface crystallization of Cs-IGZO films [23].

**Figure 5:**
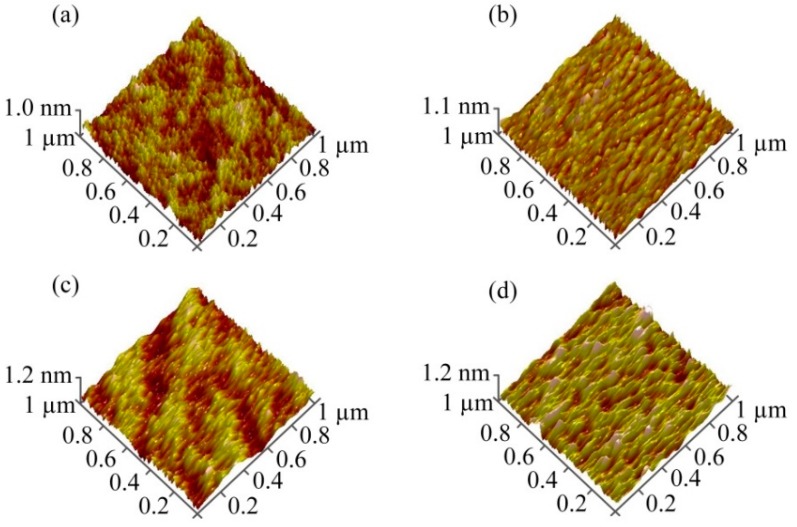
AFM images of samples of (**a**) A1, (**b**) A2, (**c**) A3, and (**d**) A4.

The surface morphology of samples A1 to A4 are measured using AFM and shown in Figure 5a–d, respectively. As observed with Figure 5, the surface morphology of samples A1 to A4 gradually changed from random arrangement to linear arrangement, which not only indicates the Cs^+^ ions adsorption could obtain a-IGZO films with regular surface structure, but also indicates that the surface structure of IGZO thin films tend to be more regular by increasing the Cs^+^ ion adsorbed degree. 

**Figure 6:**
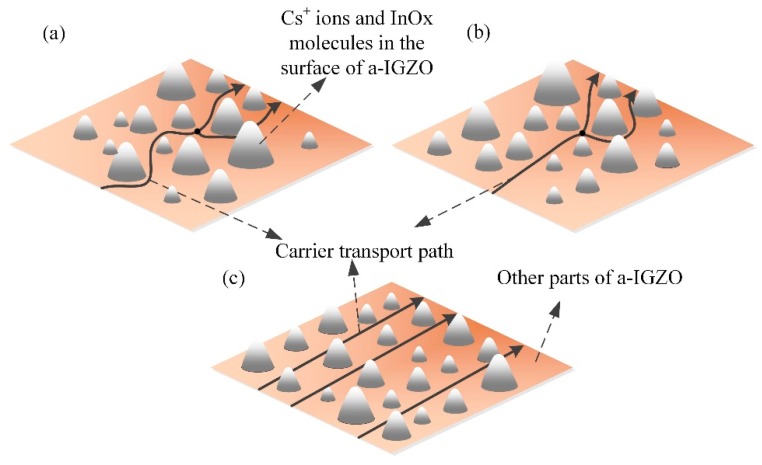
Diagram of carrier transport path in IGZO film. (**a**) barely Cs^+^ ions adsorbed IGZO film. (**b**) part of Cs^+^ ions adsorbed IGZO film. (**c**) large amount of Cs^+^ ions adsorbed IGZO film.

According to the XRD and AFM results, the movement states of carriers in Cs-IGZO films with different Cs^+^ ions adsorption are given and represented in Figure 6. Figure 6a represents the electrons transport path inside the Cs-IGZO film when the Cs^+^ ions are barely adsorbed, like sample A1. In this state, most of the metal–oxide molecules in the surface of the Cs-IGZO films are randomly arranged. Since the electrons move in the oxygen vacancies in Cs-IGZO films, the electrons need to move around each molecule, therefore the transmission paths represent the irregular way.

When the Cs^+^ ions adsorbed degree is gradually increased, some of the InO_x_ molecules on the surface of the Cs-IGZO film begin to have the regular arrangement, like samples A2. The schematic of the carrier transport path in this kind of situation is represented by Figure 6b. As adsorbed from Figure 6b, the electrons can be transmitted linearly around the regularly distributed InO_x_ molecules but are still transmitted in an irregular way around the randomly distributed other metal–oxide molecules, so their electron transport paths consist of half straight lines and half irregular lines. 

As shown in Figure 6c, with the further increase of Cs^+^ ions adsorbed degree, most of the InO_x_ molecules in the surface of the Cs-IGZO film are completely regular-arranged, like sample A3 and A4. At this time, the electrons do not need to move to bypass any molecules, so their transmission paths show a straight line. In this situation, the electrons’ transmission path will be greatly shortened, thereby improving the electrons’ transport speed efficiency, which can effectively increase the carrier mobility of the IGZO film.

### 3.3. Chemical Studies on Cs-IGZO Films

**Figure 7:**
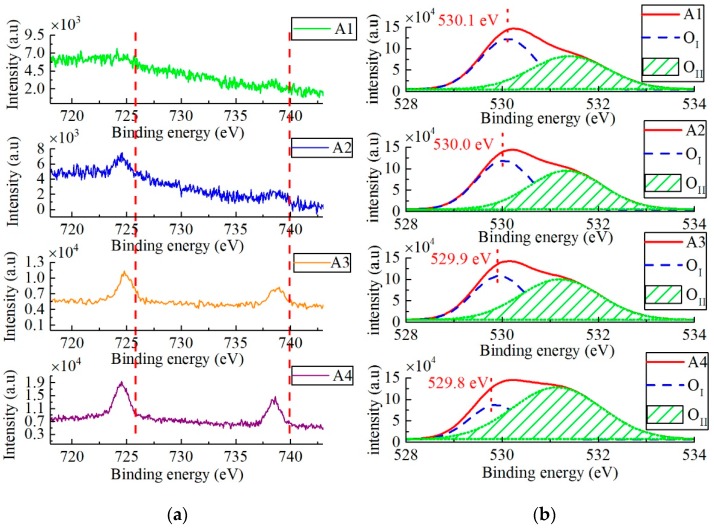
(**a**) High resolution XPS spectrum of Cs3d for sample A1 to A4. (**b**) High resolution XPS spectra of O1s for sample A1 to A4.

In order to determine the adsorbed degree of Cs^+^ ions in Cs-IGZO films, the Cs_3d_ spectrum of high-resolution XPS spectra for samples A1 to A4 are measured and represented in Figure 7a. The Cs_3d_ spectra of samples A1 to A4 are indicated by the green line, blue line, orange line, and purple line, respectively. As observed in Figure 7a, Cs_3d_ spectrum of all the four samples exhibit two distinct peaks at binding energies 724.8 eV and 738.6 eV, which represent the chemical state of Cs_3d_^2/3^ and Cs_3d_^5/2^, respectively, ensuring the presence of Cs^+^ ions. At the same time, their peak intensities increase with the increasing of CsHCO_3_ concentration, indicating that the Cs^+^ ion content in samples A1 to A4 is gradually increased. Combined with XPS analysis software, the content of Cs^+^ ions of Cs-IGZO films can be calculated. The Cs^+^ content in samples A1 to A4 is 0.49%, 0.78%, 1.21%, and 1.46%, indicating the content of Cs^+^ ions in Cs-IGZO films increases with the CsHCO_3_ concentration rising.

The O 1s spectra of samples A1 to A4 are analyzed by XPS to investigate the effect of Cs^+^ ions concentration on the characteristics of Cs-IGZO films. Generally, the O 1s spectrum of IGZO film consists of lattice oxygen (O_I_), which represents the oxygen in oxide lattices; and vacancy oxygen (O_II_), which exists in oxygen deficient. The O_I_ spectrum locates at lower binding energy with a peak of around 529.6 eV; and the O_II_ spectrum locates at higher binding energy with a peak of around 531.3 eV. According to the positions of O_I_ and O_II_ spectra, the O_1s_ spectra of samples A1 to A4 are divided into two spectra, respectively. Figure 7b shows the O_1s_ spectra and fitting curves of O_I_ and O_II_ of samples A1 to A4.

As observed in Figure 7b, the peak intensities in O_II_ spectra of samples A1 to A4 gradually increase, indicating that the oxygen vacancy concentrations of IGZO film can be significantly improved by increasing the Cs^+^ ions adsorbed degree. The area percentage under the peak of O_II_ spectrum in O_1s_ spectra (O_II_/(O_I_+O_II_)) of samples A1 to A4 are calculated to be 48.1%, 51.9%, 52.7%, and 72.3%, respectively, ascertaining that the high adsorption degree of Cs^+^ ions can generate more oxygen vacancies inside the a-IGZO film. Since the oxygen vacancy work as the donor impurities of IGZO films, this increased oxygen vacancy of Cs-IGZO films is attributed to the improvement of electrical properties of TFTs, including the negative shift of *V_th_* and the increase of *μ_FE_* and the carrier concentration.

According to the physical and chemical analysis above, the reasons for the performance improvement of Cs-IGZO TFTs are discussed. The negative shift of *V_th_* of samples A1 to A4 are attributed to the low work function of cesium and the increased oxygen vacancies of IGZO films. When Cs^+^ ions gradually adsorb in IGZO film, the low work function of cesium (2.17 eV) can gradually eliminate the Schottky barrier which exists in the interface between the source/drain electrodes and the active layer, resulting in the negative shift of *V_th_* of samples A1 to A4. When concentration of oxygen vacancy increased, the conduction band inside IGZO films gradually moved down, reducing the distance between the Fermi level and the conduction band, resulting in the negative shift of *V_th_*.

The highly improved *μ_FE_* is attributed to the increased oxygen vacancy and the regular-arranged surface structure of IGZO films. Since each oxygen vacancy generated within IGZO can provide two free electrons for the conduction band, the increased oxygen vacancies inside IGZO are usually accompanied by an improvement in the carrier concentration and carrier mobility of IGZO TFTs. The regular-arranged surface structure of IGZO films lead the carriers and can be transmitted in a straight line in the surface of IGZO film, effectively improving the carrier mobility of IGZO TFTs.

The increase of *I_OFF_* is mainly attributed to the surface single crystallization of the Cs-IGZO film by adsorbing the Cs^+^ ions. The surface single crystallization can produce the grain boundaries in the surface of Cs-IGZO. Under this circumstance, parts of electrons will flow along the grain boundary when electrons flow on the surface of Cs-IGZO, causing the growth of *I_OFF_* in Cs-IGZO TFT. As the degree of crystallization of Cs-IGZO surface increases, more electrons can flow between the grain boundaries, resulting in the improvement of *I_OFF_* in Cs-IGZO TFTs.

**Figure 8:**
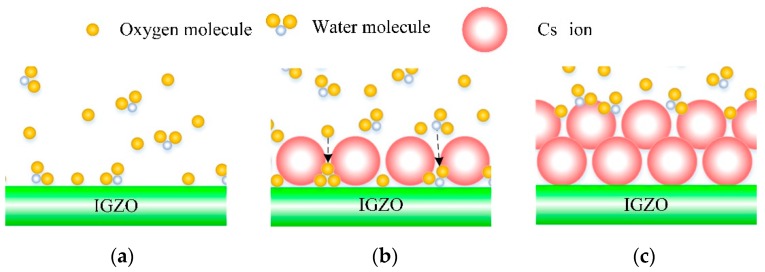
Schematic diagram of adsorption of H_2_O and oxygen molecules in the surface of Cs-IGZO and amorphous InGaZnO_x_ (a-IGZO) films. (**a**) barely Cs^+^ ions adsorbed IGZO film. (**b**) part of Cs^+^ ions adsorbed IGZO film. (**c**) large amount of Cs^+^ ions adsorbed IGZO film.

The reason for the optimized stability of Cs-IGZO TFTs is analyzed and shown in Figure 8.

Figure 8a is the schematic which shows the adsorbed state of oxygen molecules and water molecules in air on the bare Cs^+^ ions adsorbed IGZO film. In this situation, oxygen molecules and water molecules can be adsorbed in the surface of the IGZO film without any barrier, resulting in the positive shift of *V_th_* of IGZO TFTs. When Cs^+^ ions are gradually adsorbed into IGZO, the Cs^+^ ions can block the contact of IGZO with molecules in the air, greatly reducing the adsorption of oxygen molecules and water molecules on the surface of IGZO, thereby effectively improving the stability of IGZO TFT, as shown in Figure 8b. However, since Cs^+^ ions have a large ionic radius, oxygen molecules and water molecules can pass through the interspace between the Cs^+^ ions to adsorb in the IGZO film, therefore small amount of Cs^+^ ion adsorption cannot completely isolate the contact between IGZO and molecules in air. As observed in Figure 8c, the oxygen molecules and water molecules cannot pass through the Cs^+^ ions barrier to adsorb in the surface of IGZO film when a large number of Cs^+^ ions are adsorbed in IGZO film. In this state, the IGZO TFT has superior stability, and its stability cannot be improved as the Cs^+^ ions concentration increases. Combined with the electrical properties of samples A1 to A4, it can be concluded that sample A3 is the optimized Cs-IGZO TFT which has the optimized performance.

### 3.4. Comparison between Cs-IGZO TFT and Annealed IGZO TFT

**Figure 9:**
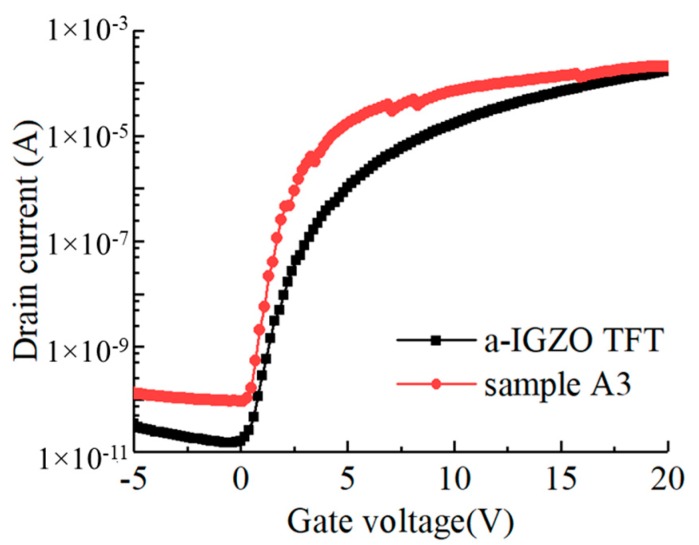
Comparison of electrical properties between sample A3 and annealed IGZO TFT.

The performance of the optimized Cs-IGZO TFT (sample A3) and a-IGZO TFT which was annealed at 400 °C for 30 min in N_2_ atmosphere is compared. The transfer properties of sample A3 and annealed a-IGZO TFT are represented in Figure 9, and the *V_th_*, *μ_FE_*, and *S.S* are calculated and represented in Table 3. As observed in Figure 9 and Table 3, sample A3 has a similar *V_th_* to that of annealed a-IGZO TFT which is 0.2 V, and its *μ_FE_* and *S.S* are superior to that of a-IGZO TFT, indicating the transfer properties of optimized Cs-IGZO TFT are superior to that of annealed IGZO TFT.

**Figure 10:**
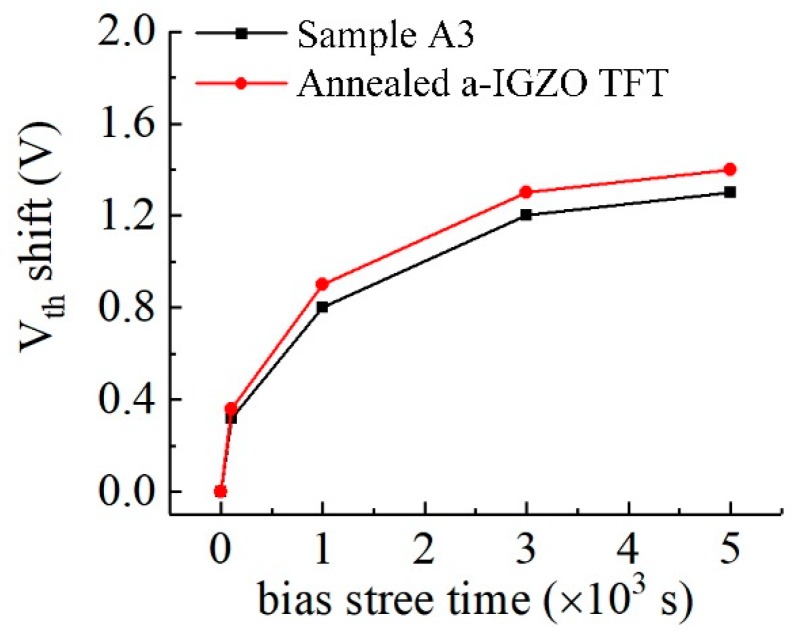
*V_th_* shifts of optimized Cs-IGZO TFT and a-IGZO TFT.

The *V_th_* shifts under gate bias of sample A3 and annealed a-IGZO TFT are calculated and shown in Figure 10. 

As observed in Figure 10, it is obvious that the Δ*V_th_* of sample A3 (1.3 V) is smaller than that of annealed a-IGZO TFT (1.4 V). These results indicate that the Cs-IGZO TFT fabricated at a low temperature has the potential to replace the annealed a-IGZO TFTs, and is suitable for use in flexible substrates.

## 4. Conclusions

In summary, Cs^+^ ions were adsorbed into IGZO film by CsHCO_3_ solution immersion method at low temperature, and had the benefit of improving IGZO TFTs performance. Four kinds of CsHCO_3_ concentrations were selected to optimize the transfer property and stability of Cs-IGZO TFTs. According to the electrical measurements of Cs-IGZO TFTs, it was found that the Cs-IGZO TFT with CsHCO_3_ concentration of 2% in water solution had the optimized electrical properties, including high *μ_FE_* of 18.7 cm^2^ V^−1^ s^−1^, small threshold voltage shifts of 1.3 V, and low OFF current of 0.8 × 10^−10^ A. These superior performances of optimized Cs-IGZO TFT were attributed to the change of surface structure and oxygen vacancy concentrations of IGZO film, by appropriate Cs^+^ ions adsorbed into IGZO film. Compared with the traditional a-IGZO TFTs fabricated through annealing process, the optimized Cs-IGZO TFT had superior mobility and comparable device stability, which might be applicable to future flexible electronics.

## Figures and Tables

**Table 1:** Name of Cs-IGZO films.

Name of Cs-IGZO Film	A1	A2	A3	A4
**Concentration of CsHCO_3_ Solution (% mol/L)**	0.5	1	2	3

**Table 2:** *V_th_* and *S.S* value of samples A1 to A4.

Parameter/Sample	*V_th_* (V)	*μ_FE_* (cm^2^ V^−1^ s^−1^)	*S.S* (V/decade)
A1	7.9	8.6	0.28
A2	5.7	13.1	0.25
A3	0.2	18.7	0.23
A4	−1.7	21.5	0.22

**Table 3:** Electrical properties of optimized Cs-IGZO TFT and a-IGZO TFT.

Samples/Parameter	*V_th_* (V)	*μ_FE_* (cm^2^ V^−1^ s^−1^)	*S.S* (V/decade)
Cs-IGZO TFT	0.2	18.7	0.23
a-IGZO TFT	0.2	12.6	0.24
